# Correction: Zhang et al. Biodegradable Starch/Chitosan Foam via Microwave Assisted Preparation: Morphology and Performance Properties. *Polymers* 2020, *12*, 2612

**DOI:** 10.3390/polym14091691

**Published:** 2022-04-21

**Authors:** Xian Zhang, Zhuangzhuang Teng, Runzhou Huang, Jeffrey M. Catchmark

**Affiliations:** 1 Co-Innovation Center of Efficient Processing and Utilization of Forest Products, College of Materials Science and Engineering, Nanjing Forestry University, Nanjing 210037, China; shinezhang17@163.com (X.Z.); RowanZJ@163.com (Z.T.); 2 Department of Agricultural and Biological Engineering, The Pennsylvania State University, University Park, State College, PA 16802, USA

## Addition of an Author

Jeffrey M. Catchmark was not included as an author in the original publication [1]. The corrected Author Contributions Statement appears here. The authors apologize for any inconvenience caused and state that the scientific conclusions are unaffected. This correction was approved by the Academic Editor. The original publication has also been updated.
